# A mixed method study on the impact of living with spinal muscular atrophy in Malaysia from patients’ and caregivers’ perspectives

**DOI:** 10.1186/s13023-022-02351-4

**Published:** 2022-05-16

**Authors:** Gaik Siew Ch’ng, Karina Koh, Azlina Ahmad-Annuar, Fahisham Taib, Cha Ling Koh, Edmund Soon Chin Lim

**Affiliations:** 1 Department of Genetics, Hospital Pulau Pinang, Ministry of Health, Jalan Residensi, 10990 George Town, Penang Malaysia; 2 Clinical Research Centre, Hospital Kuala Lumpur, Ministry of Health, Jalan Pahang, 50586 Kuala Lumpur, Malaysia; 3 Department of Biomedical Science, Faculty of Medicine, University of Malaya, 50603 Kuala Lumpur, Malaysia; 4 Department of Paediatrics, Hospital Universiti Sains Malaysia, 16150 Kubang Kerian, Kota Bharu, Kelantan Malaysia; 5 HELP University, Wisma HELP, Jalan Dungun, Bukit Damansara, 50490 Kuala Lumpur, Malaysia; 6 Persatuan Kebajikan Ceriajaya Kuala Lumpur Dan Selangor (WeCareJourney), Suite 105, MBE Uptown, No 54, GF, Jalan SS21/58, Damansara Uptown, 47400 Petaling Jaya, Selangor Malaysia

**Keywords:** Spinal muscular atrophy, Malaysia, Persons with spinal muscular atrophy, Caregiver, Neuromuscular disorder, Progressive muscle weakness, Psychosocial

## Abstract

**Background:**

Spinal Muscular Atrophy (SMA) is a rare, recessively inherited neuromuscular disorder that causes progressive muscle weakness. There is a low degree of awareness about SMA amongst the public and healthcare providers, which may impact the perception of the disease and its proper management. To understand how this lack of awareness may have affected diagnosis, care and support for SMA patients and their caregivers, this study aims to investigate the impact of SMA on the lives and daily activities of SMA patients and their caregivers in Malaysia.

**Methods:**

Nationwide recruitment was carried out via invitations coordinated by a local SMA advocacy organization. A mixed method cross-sectional study consisting of a self-administered questionnaire followed by in-depth interviews (IDIs) and focus group discussions (FGDs) was conducted. The interview sessions were audio-taped, and verbatim transcripts analyzed thematically.

**Results:**

Participants reported feeling stressed, anxious and depressed. There were issues with delayed diagnosis, lack of information from healthcare professionals about the disease progression, and limited access to supportive services like physiotherapy. Participants expressed their concerns living with self-doubt and turmoil with having to modify their lifestyles, relationships with family and friends, and challenges with educational and career opportunities. Various themes of their hopes for the future touched on having access to treatment, clinical trials, holistic care for symptom management, as well as improving infrastructure for disability access.

**Conclusion:**

This study, to the best of our knowledge represents the first comprehensive study on SMA in South East Asia, highlights a plethora of issues and challenges experienced by persons with spinal muscular atrophy (PWSMA) and their caregivers in Malaysia, from the point of SMA diagnosis and throughout the management of care, in addition to the deep psychosocial impact of living with SMA. The significant findings of this study may contribute to a better understanding among stakeholders to make improvements in clinical practice, the education system, the work environment as well as holistic care support and society at large.

## Background

Spinal muscular atrophy (SMA) is a rare, recessively inherited neuromuscular disorder caused by deletions or mutations in the survival motor neuron 1 gene (*SMN1*), and the severity is modified by the number of *SMN2* copies. The estimated prevalence in Europe ranges from 1 in 3600 to 16,000 [1] and world-wide incidence averages 1 in 10,000 livebirths [2]. In Malaysia, the exact prevalence is unknown but the estimated incidence is 1 per 20,000 livebirths [3]. The spectrum of disease is divided into various types by the level of weakness, according to the ‘best achievable motor function’ in their life time. The clinical type of SMA is defined by the age of the onset and the severity of the disease, from type 0 (most severe) to type 4 (adult onset) [2].

There is no cure for SMA, but FDA-approved disease modifying therapies such as Nusinersen (Spinraza), Zolgensma (Onasemnogene abeparvovec) and Risdiplam (Evrysdi) can delay disease progression. This chronic and debilitating disease places an enormous strain on the families affected which reported a lower quality of life score and higher stress levels [4, 5, 7], However, these viewpoints came from individuals living in upper income countries, and there is limited information about the perspectives in low-middle income countries where healthcare and supportive resources are scarce.

To the best of our knowledge, there have not been any studies exploring the impact of the disease on persons with spinal muscular atrophy (PWSMA) and their families in South East Asia, a region with over 677 million individuals from diverse ancestries. These communities with their multi-ethnic and multi-religious perspectives represent a rich resource to further understand their experiences, challenges and coping strategies which may be unique compared to Western communities. This study is the first South East Asian study to comprehensively investigate the impact of SMA on the lives and daily activities of SMA patients and their caregivers.

## Methods

### Recruitment and sampling

PWSMA and caregivers were recruited through advertisement among members of a local SMA advocacy organization, WeCareJourney. Invitations were sent via email or other social media platforms. Inclusion criteria for the participants were; a) persons who were diagnosed with any type of SMA, either by clinical or genetic tests; b) caregivers of individuals with SMA who are either a family member or non-family member who provides a minimum of 4 h per day of care with at least one activity of daily living (ADL) [6].

### Data collection and analysis

Ethical approval for this study was obtained from the Malaysian Research and Ethics Committee (NMRR-18-3248-42367). A mixed method cross-sectional study consisting of a self-administered questionnaire followed by in-depth interviews (IDI) and focus group discussions (FGD) were  used to collect quantitative and qualitative data between April to December 2019. Triangulation was performed to interpret the rich data findings from the study.

The questionnaires were adapted from Voice of the Patient report, Cure SMA 2018 and Qian et al. 2015 survey that looked into the experiences and needs of PWSMA and their caregivers [5, 7], with consent obtained from the original authors for the use and local adaptation of the tools. The adapted ‘MySMA Quantitative’ questionnaire covered aspects concerning participants’ demographics, diagnostic history of SMA activities of daily living (ADL), and information about disease management. Both the ‘MySMA Quantitative’ and ‘MySMA Qualitative’ [7] surveys were translated into the national language (Malay) and Mandarin, and validated using standard methods. Semi-structured interviews using questions from ‘MySMA Qualitative’ document explored the participants’ views, either via IDI or FGD regarding experiences reaching a diagnosis, the impact of the disease, concerns, and hopes for the future. The interviews were conducted by a team of researchers in the native languages of the participants. Each interview was audio-recorded and transcribed verbatim for thematic analysis. Transcripts were read repeatedly to allow familiarity with the qualitative data. We utilized the grounded theory principle with deductive and inductive types of coding, and codings were based on the analysis of participants’ responses. These codes were analyzed by two separate researchers combining emerging themes from the deductive and inductive processes. The salient findings across all interviews were synthesized and the main unifying themes that characterized the participants’ experiences were captured.

## Results

A total of forty-two (42) individuals participated in the quantitative survey (Table 1), of whom 13 (31%) were PWSMA and 29 (69%) were caregivers; and of which 62% were female. Several ethnic groups (based on self-reported ethnicity) were represented, including Malay Malaysian (69%); Chinese Malaysian (24%), Indian Malaysian (5%) and a foreign national who was a caregiver (2%). The majority of PWSMA were above 19 years old (84.6%), with the youngest, an eleven-year-old Type 3 PWSMA, and overall, 69% were Type 2, with the remaining 31% Type 3. The caregivers who participated cared for PWSMA of varying types; Type 1 (28%), Type 2 (38%) and Type 3 (34%). All but one of the PWSMA received some form of education from primary to tertiary level, where one PWSMA did not receive any formal education. Of the caregivers, (27/29; 93%) received secondary or tertiary education, one without any formal education, and another did not respond.

**Table 1:** Characteristic of 42 study participants

Characteristics	PWSMA N = 13 (31%)	Caregivers of PWSMA N = 29 (69%)
*Gender*		
Male	5 (38)	11 (38)
Female	8 (62)	18 (62)
*Ethnicity*		
Malay	7 (54)	22 (76)
Chinese	5 (38)	5(17)
Indian	1 (8)	1 (3)
Others	0	1 (3)
*Age (years)*		
10–19	2 (14)	–
20–29	9 (69)	2 (7)
30–39	1 (8)	10 (34)
40–49	1 (8)	9 (31)
50 and above	0	8 (28)
*SMA type*		
Type 1	0 (0)	8 (28)
Type 2	9 (69)	11 (38)
Type 3	4 (31)	10 (34)
*Highest formal education*		
No formal education	1 (8)	1 (3)
Primary	4 (31)	0 (0)
Secondary	2 (15)	12 (42)
Tertiary	6 (46)	15 (52)
Did not respond	–	1 (3)

From the quantitative survey, more than half of the PWSMA (n = 7/13; 54%) reported having to cope with mental health issues, acknowledging that they felt at least one of the following emotions; stress, anxiety or depression (Table 2). Out of these, PWSMA with Type 3 were the most affected (n = 3/4; 75%), while 56% of PWSMA Type 2 (n = 5/9) reported having some mental health issues. In addition, approximately a third of PWSMA (n = 4/13; 31%) lamented having feelings of social isolation. Meanwhile for the caregivers, (n = 24/29; 83%) reported having the same issues with stress, anxiety and depression, with caregivers of PWSMA Type 1 being most affected (n = 10/10; 100%). Feelings of social isolation were reported in 3 out of 29 caregivers (10%).

**Table 2:** Psychosocial impact of living with SMA

Psychosocial impact of SMA	PWSMA N = 13 (%)	Caregivers of PWSMA N = 29 (%)
*Mental health*		
Feel stressed	7 (54)	16 (55)
Feel depressed	5 (38)	6 (21)
Feel anxious	5 (38)	22 (76)
Feel socially isolated	4 (31)	3 (10)
Lack independence	6 (46)	8 (27)
*Physical issues*		
Lack mobility independence	7 (54)	24 (83)
Unable to transfer	7 (54)	17 (59)
Limited physical activities	7 (54)	16 (55)
Unable to go to toilet oneself	6 (46)	15 (52)
Unable to turn in bed	5 (38)	11 (38)
Unable to care for personal hygiene	4 (31)	15 (52)
Unable to dress self	4 (31)	11 (38)
Unable to feed oneself	3 (23)	5 (17)
*Psychosocial impact*		
Lost job	2 (15)	4 (14)
Troubled relationships	3 (23)	2 (7)
*Disruption to career/education*		
Unable to attend work/school	4 (31)	8 (28)
Diminished social activities	4 (31)	6 (21)

As expected for a neuromuscular disorder, both PWSMA and caregivers voiced issues coping with the physical limitations imposed by SMA. The highest rated issues for the PWSMA were the lack of independent mobility (54%), being unable to transfer from one position to another (54%), and challenges with physical activities (54%). This was followed by being unable to go to the toilet by themselves (46%), being unable to turn in bed (38%), and personal hygiene/self-grooming (31% each), and not being able to feed themselves (23%).

A similar trend across the different aspects was seen with the caregivers, however comparatively, it appeared that the caregivers felt greater issues with the PWSMA lack of independent mobility (PWSMA: 54% vs caregivers: 83%) and ability to attend to their own personal hygiene (PWSMA: 31% vs caregivers: 52%).

Interestingly, the caregivers' perspective concurred with the PWSMA about which daily activities are most limited by SMA, also noting that the limited ability to mobilise independently (83%), cope with physical activities (55%) or transfer themselves (58%) to be the most challenging. Additionally, a large number of caregivers (54%) viewed that the difficulty of PWSMA to attend to personal hygiene was also a trying issue.

Out of the thirteen PWSMA who participated in the survey, seven consented to continue on with in-depth interviews; four women and three men (age range 19–42 years). Five of them have Type 2 SMA and two others have Type 3 SMA. Additionally, twenty-three caregivers (parents to PWSMA) also consented to be  interviewed either in FGDs or IDIs, where the age of their (surviving) children ranged from 2 to 23 years old. All recordings of the interviews were transcribed verbatim, and as the interviewers all asked a set list of questions (ranging from diagnosis, management of the symptoms, supportive services, psychosocial impact and others), trained researchers were able to analyse the conversations to detect common themes that appeared.

Overall, the interview sessions uncovered several key common themes from both PWSMA and their caregivers (summarised in Table 3 with associated quotes).

**Table 3:** Summary of FGD and IDI findings

Topic	Emerging themes
Experiences Related to Diagnosis	**a) Long journey to final diagnosis** Many caregivers shared how difficult it was to get their child diagnosed. For example, LL faced a delayed in reaching a diagnosis of SMA and a long arduous journey filled with confusion and uncertainty, often met by dismissive or even insensitive health professionals *“I think about for one year plus we were searching for treatments, for one whole year! To our disappointment we can't find anyone, nobody seemed to know. Many doctors do not know actually. Very sad to say.”* (Mrs LL to 20-year-old daughter, SMA Type 2) **b) Dismissive healthcare professionals** “*Doctors lack empathy and did not offer much help. The doctor said ‘eh, you know or not your child ah, can’t live long. I can only give you a period of a maximum of 2 years.”* (Mr BB, father to 2 deceased SMA Type 1 children) **c) Acceptance and coping with the diagnosis** Caregivers expressed various emotional responses knowing the outcome and diagnosis of the condition. This was stressed by Mrs XX on her poor preparation and guilt *“There is a feeling of guilt, like there is something with my pregnancy, because I could not comprehend at that time. Actually, at that time, we do not understand the seriousness of the diagnosis… in one to two years I [still] cannot accept [the diagnosis].”* (Mrs XX, mother of 2 SMA Type 2 children) *“We have come to accept it, a lot of people see the way we provide for my child and asks if I am tired? I said yes, it is definitely tiring, then they ask if I am angry, I say I am not angry at all.”* (Mr AA, father of 21-year-old, SMA Type 3) PWSMAs, on the other hand, grew up knowing they are different from other children and understood the information at a much older age. Most of them echoed similar experiences of the caregivers for being misdiagnosed after many hospital visits. Additionally, various factors such as type of upbringing and religious faith play key roles for PWSMA in accepting and coping with the diagnosis *“I think growing up, it was, kind of complicated because I always wondered why am I weaker than others, I thought there is something wrong, I didn’t really understand, like for example why can’t I run like the other boys? Why they are faster than me? Why do I get tired so fast?*” (Mr G, 22 years old, SMA Type 2) *“Whenever I recovered from a febrile illness, I somehow seemed to lose something within me. I could no longer able to lift my hands up. Previously I was able to hold my books, remove them from my school bag; but when i got older, those similar sized books were much heavier; I needed my classmates’ assistance to put my books from my bag to the table, and eventually I could no longer do it by myself.”* (Ms D, 42 years old, SMA Type 2) *“I didn’t know about SMA, until my 15-year-old sister had her 1st hospitalisation (and her last)* [younger sister succumbed to the illness]*, then I googled about SMA… but nothing really changes. Everything is still the same (after knowing I have SMA) because my parents taught us to be positive.”* *(Ms B, 23 years old, SMA Type 3 who only ‘found out’ about SMA when she turned 18)* *“Because my parents had neglected my medical condition for too long… the pain was no longer bearable… I was brought up with the belief that I was lazy… because they always tell me that you are lazy that’s why you can’t lift your arms, can’t walk, can’t balance, and if you fall down, they think, I was doing it on purpose…* feeling negative the whole time. S*o, after I knew about the diagnosis, I started researching, learning and teaching myself about SMA. I was diagnosed with depression when I was 20 years old and was suicidal; and needed to be on medication* *(Ms. A, 23 years old, SMA Type 3, who grew up with a single mother who suffered from depression and schizophrenia)* *“I grow up with strong faith, religion, so, yeah, I rely on that a lot.”* (Mr G, 22 years old, SMA Type 2)
Psychosocial impact of SMA	Among the PWSMA, the key themes that emerged were juxtaposed between some feeling self-doubt, loneliness and depression where else some say SMA brought their family closer together **a) Self-doubt & inner turmoil** “*I grew up always questioning ‘Why am I weaker than others?’, ‘Why can’t I run like the other boys?’, ‘why do I always fall down? What am I? Am I special? I realised I am just different from the rest.”* (Mr G, 22 years old, SMA Type 2) **b) Depression** *“I have a lot of things to worry about, because I don’t have support from my family, most of the time I am alone, especially in dealing with problems, just by myself, no one to turn to, so it can be quite difficult, sometimes I feel really tired, and the suicidal thoughts would come again, and repeat itself.*” (Ms A, 23- year old, SMA Type 3) **c) Unsupportive schooling environment & bullying** *“In primary school, I think that was the hardest time of my life because I faced many challenges in primary school like for instance my class room was on the first floor and the teachers weren’t very, I would say supportive, like my parents talked to them but still they didn’t put class downstairs.”* (Mr G, 22 years old, SMA Type 2) *“There was an incident of bullying because of my condition, I couldn't lift my head up. One of my classmates pushed my head down and left me there until another classmate called my helper in to help me and lift my head up.”* (Mr E, 29 years old, SMA Type 2) **d) Fostered meaningful relationships** *“This condition makes me actually closer to my family, because I rely on them for help, and by relying on them for help I actually form a good bond, a good relationship, whether I like it or not, ha-ha, I think if without this I will probably be on my own, you know, probably will not be spending much time with them.”* (Mr G, 22 years old, SMA Type 2) *“Hang out every other day. Ah…amazing people. They are all either extremely creative or extremely intelligent. We do the whole dinner table conversations with like usually six to a lot of people.”* (Ms C, 32 years old, SMA Type 2) Among caregivers, many struggled with their changing lifestyles, stress, anxiety, burnout and financial burdens **a) Changing lifestyles** *“For family occasion, kenduri(ceremony), weddings, I would reject invitations from all my relatives. I never went.”* (Mrs PP, mother of deceased SMA Type 2) *“I tend to cut down on all my social activities. So family is everything. We take him as much as possible for family outings. Furthermore, going out as a couple has become a rare occasion as at least one parent has to stay at home in the evening.”* (Mr CC, father to 23-year-old SMA Type 2) **b) Stress, anxiety and burn out** *“At times we become angry. Then we calmed down. The test from God is great.”* (Mr HH, father to 2 surviving SMA children) *“Sometimes we can’t take care of our daughter, because we are paying more attention to our son and we do feel bad.”* (Mr CC, father to 23-year-old SMA Type 2) **c) Financial burdens** *“Because I work as a lorry driver with a daily-paid salary (and my wife is not working), whenever my son gets admitted into hospital, I have to take leave; if I don’t work, then I have no income.”* *(Mr NN, father* of two deceased SMA Type 1) *“From the aspect of cost because oxygen machine, suction machine all that we bought on our own. We did not receive any help from anywhere.”* (Mrs NN, mother of two deceased SMA Type 1)
Worries and concerns	When discussing worries and concern, independence is a recurring theme of all the interviews. Participants also discussed social discomfiture and the lack of infrastructure for the differently abled in Malaysia **a) Functionally independent** *“I worry the most right now would be not being able to live independently in the future, yes, because I know my parents will not always be there for me, so wondering that if I will be able to find a way to live independently you know, when my parents are not there, so that’s what worries me the most.”* (Mr G, 22 years old, SMA Type 2) **b) Financially independent** *“His condition and that would affect his independence, and of course, there’s also another thing, affordability, affordability means, what would be his financial situation at that point of time?”* (Mr CC, father to 23-year-old SMA Type 2) **c) Social discomfiture** *“Other family members, they just see that my sister and I can’t walk, can’t do things. Even the well-educated ones, a cousin who is a doctor, when we tried to explain about SMA, they don’t want to know.”* (Ms B, 22 years old, SMA Type 3) *“The infrastructure in Malaysia is really incomplete, because you have these disabilities pathways which suddenly break off in the middle, and you don’t know where that leads to. A huge problem with the lifts, if they break down, if people are trying to get in the lift, even though they see there is a wheelchair there, they don’t wait for me [to get out] so I just drag myself to the side, and I just let them all go in there, and I will just wait for the next one. The facilities are not maintained, the inconsideration of people parking in disabled spots.”* (Ms A, 23-year old, SMA Type 3)
Future hopes and wishes	Future hopes and wishes for the future expressed by the participants can be grouped under several themes: **a) Access to treatment** In terms of new treatment, all PWSMA welcome anything which will enable them to improve their functional abilities and/or slow down the deterioration of their condition *“Anything which enable me to gain back ability to take care of myself or it helps to slow down deterioration rate of my condition.”* (Ms D, 42-year-old SMA Type 2) *“My suggestion and top priority for the government is bring in Spinraza*. *And put aside a certain allocation, you know, every year for the SMA patients.”* (Mr DD, father to 11-year-old SMA Type 3) **b) Government to improve medical care services to ensure holistic care post diagnosis** There is a need to provide support and assistance to families such as access to palliative care, mental health and counselling services, equipment rental, respite care services, etc *“I think palliative care is very important. The palliative team needs to come in and talk to the parents. Even if I know my child is going to pass on, but during this period, what is the best I can do?”* (Mrs SS, mother to 8-year-old SMA Type 1c) *“Counselling for grieving parents, I mean if the parents can talk to somebody, you know, a professional counsellor. To talk to a person who is totally impartial, and a professional to listen, you know. I think it’s important.”* (Mrs SS, mother to 8-year-old SMA Type 1c) *“Machines like cough assist is required. To help them cough. Others are machine [for] monitoring and nebulizer, so parents need information to use the equipment.”* (Mrs XX, mother of 2 SMA Type 2 children) *“We are very tired! And, if the adults, or the caregivers are not taken care of, it doesn’t only affect the child with SMA but it affects the whole family. The relationship for their children also.”* (Mrs SS, mother to 8-year-old SMA Type 1c) **c) Government to improve accessibility and be inclusive** The hope for improving the life of children and adults with SMA, as well as for their caregivers are passionately voiced in all IDIs and FGDs *“I think the number one thing would be to make places more accessible. Some of us on wheelchair, some of us use stretchers, but I think it’s important to make every place accessible, so as to make our life easier. They have no idea how a small thing such as a ramp can make such a big difference.”* (Mr G, 22 years old, SMA Type 2) **a) Policymakers to ensure a clear and committed policy direction** *“If you have a system that appreciates you as a human being you won't be driven to the corner by people who don't know what or who you are. There will be no stigma because stigma has been eradicated by simple laws against discrimination. So, law is very important.”* (Ms G, 32- year old, SMA Type 2)

### Receiving the diagnosis from the caregivers' perspective

The first time most caregivers had heard about the medical condition was when their child was diagnosed. The length of time to achieve this diagnosis ranged from one month for the severe type 1 to 28 years for a 42-year-old PWSMA with Type 2, reflecting on the long, anxious period that the parents went through. For first time parents, in hindsight, they reported noticing some warning symptoms like difficulty with suckling, lack of fetal movement, turning blue or delays in physical milestones like sitting up, but experienced alternative diagnosis/reasons from healthcare professionals. For caregivers with previous children, they also noticed early signs especially since they would have experienced ‘typical’ newborn behaviours previously, but they all shared that they would not have suspected their child to have a genetic neuromuscular disorder, given no family history, and general lack of awareness of the condition. Experiences with dismissive clinicians or those who did not offer much support post-diagnosis were common. Feelings of shock, deep sadness and grief, uncertainty for the future, high levels of stress and anxiety were commonly mentioned by all the caregivers. Many turned to the internet to look for more information about SMA, and for many, this led to feelings of anguish and concern about the disease prognosis where the words ‘no cure’ was hard to accept. They also immediately reached out to connect with family support groups both locally and internationally. In this regard, while there are now active local SMA support groups who are easily reachable through the internet and social media platforms, for caregivers whose children were diagnosed before 2016, there would not have been any local family support groups and associated supportive care, which coupled with poor internet connectivity, would have caused the caregivers to feel alone and completely lost in understanding how to manage the symptoms. In addition, it was emotionally taxing for families who had previously encountered a traumatic death experience of their first child, and having to come to terms with having another child with SMA, compounded with the uncertain disease trajectory and foreknowledge of premature death.

### Understanding, and coming to terms with the diagnosis from a PWSMAs' perspective

All of the PWSMA interviewed were of Type 2 and 3 with median length of duration to reach a diagnosis of 6 years (ranged 1–28 years). All PWSMA expressed gradually losing control in managing daily tasks, especially the inability to recover muscle strength fully to baseline and further regression following a febrile illness (quote Ms. D, Table 3).

Many expressed the realisation that they were different around school-going age (quote by Mr. G, Table 3), or when comparing themselves with older siblings.

Caregivers’ mental health, support and home environment play a huge role in influencing how PWSMA view and accept their diagnosis of SMA; exemplified by the quotes in Table 3; from growing up with very positive thinking parents, to overprotective parenting style to outright parental neglect.

### Psychosocial impact on PWSMA and caregivers

The key stressors for caregivers were chronic worrying about the PWSMA under their care, exhaustion, changing lifestyles and coping with financial issues (Table 3 quotes). Many caregivers of SMA were lost in navigating the complex, frustrating, and fragmented array of services, especially transition of medical care from childhood to adulthood. They often withdrew from social engagements due to concerns of health risks for their child, however, despite this, the majority of the caregivers (90%) did not feel socially isolated, recounting help from other family members and close friends.

All PWSMA had varying schooling experiences. Three PWSMA recounted the experience of being discriminated against or bullied in school such as being teased for walking ‘funnily’ and presumed to be mentally challenged and put in remedial class. Few others were blessed with well supported schooling environment and understanding from school authorities who provided critical aid and comfort which helped them cope with the demands and enjoy school/college/university-life.

PWSMA often also reported facing discrimination during work recruitment and felt that they were not given equal job opportunities, in addition to lack of accessibility to disable-friendly toilets and not accommodating flexible working hours. This negatively impacted their earning abilities, making them reliant on caregivers and could not be fully independent financially.

For caregivers, the need for frequent hospital visits occasionally caused friction at work and loss of potential job opportunities, thus further impacting their finances. (quotes in Table 3).

### Worries and concerns

Both caregivers and PWSMA lamented the loss of what they considered a ‘normal way of life’, and had feelings of disappointment, loss of motivation, helplessness, anxiety and uncertainty for their/their children’s future; with concerns about the lack of independence/being dependent on others being the recurring theme among all interviewees. Many also discussed social discomfiture and the lack of infrastructure for the differently abled in Malaysia which further compounded on their mental wellbeing (quotes in Table 3).

### Future hopes and wishes

Several themes emerged when discussing about future hopes and wishes; with the most resounding one being the call for available treatment to be made accessible in Malaysia. All PWSMA would welcome any treatment which would improve their functional abilities and/or slow down the deterioration of their condition. Almost all of the participants stressed for the government to improve medical care services to ensure holistic care can be given to the SMA community post-diagnosis including access to palliative care, mental health and counselling services, equipment rental and respite care services. Other than that, many hoped that the government can continue to improve accessibility and inclusivity for the differently abled in Malaysia. (quotes in Table 3).

## Discussion

The main aim of this study was to explore the impact of living with SMA in the Malaysian context. Despite SMA being one of the most common inherited neuromuscular disorders, to the best of our knowledge, this is the first report on SMA perspectives in PWSMA and their caregivers in South East Asia. When compared with studies that have been done in other populations across the world, including in China [8], Europe [9, 10], South and North America [11], this study aims to fill this significant gap in our knowledge of the issues and challenges faced by PWSMA and their caregivers in Malaysia, where economic, cultural and religious nuances may give rise to different challenges and influence perspectives compared to the reported studies in Western countries. It is especially critical to understand more about these aspects in under-reported populations given the dynamic and fast-changing landscape for disease modifying therapies in SMA. Findings in our Malaysian families also offer far-reaching insights about the lives of SMA families in other countries such as Singapore, Brunei and Indonesia due to close ethnic and socio-economic relations. In keeping with other published studies, this survey was performed in partnership with patient advocate groups, healthcare professionals and academics. There is no SMA registry in Malaysia, thus study participants were recruited through advertisements by the patient support group, and were of a diverse sociodemographic group from urban and rural areas as well as including adult and child PWSMAs.

The psychosocial impact of SMA is clearly profound as more than half of participants from this study reported to experiencing stress, and majority of caregivers experiencing anxiety; similar to earlier studies conducted which reported the high emotional costs of living with SMA [12, 13]. Despite this, only 30% of PWSMA and 10% of caregivers reported to feeling socially isolated with many citing good social supports from their family and friends as their source of strength and comfort, similar to a study by Lamb & Peden which reported that all their participants described having a strong and good network of family, friends, and social support to be their pillar of strength [14]. It is also important to note that despite having substantial mental health issues, only one caregiver sought professional help, and this may be due to cultural stigma surrounding receiving psychological counselling, and the Asian mentality of keeping emotions silent. Mental health care is still a largely unmet need among this community, as highlighted in a similar study [15]. The mental health repercussions arise due to unavailability of respite care in the community, an essential system to relieve parents of their duties temporarily and compassionately. This is a stark contrast to the services that are available in other countries which reported that among caregivers to PWSMA; 45.7% had received respite care before due to exhaustion and about a third of both PWSMA and their caregivers have received counselling services [10, 16]. Despite the challenges and setbacks, this study’s participants reported feeling solidarity and camaraderie from support groups. Support groups provide mutual aid, information-sharing and similar goals, which empower SMA families to be more committed and confident in facing challenges [15].

Higher percentage of caregivers (76%) were reported to experience anxiety compared to PWSMA (38%). This feeling of anxiousness would have begun from the time of noticing differences in the child in terms of their behaviour and muscle strength, and continued due to the healthcare professionals’ *‘Wait and see’* approach, which left them without answers for prolonged periods of time. This anxiety was also driven by the lack of awareness and recognition of SMA by many parties involved [14, 17]. For families who had previously faced the death of a child, the uncertain disease trajectory for the other PWSMA family member compounded by foreknowledge of premature death, is emotionally taxing as reported in previous studies regarding the emotional toll of being a SMA caregiver [17]. Delays in diagnosis was experienced by all of the first-time caregivers, wide ranging from 3 months to 28 years (median of 6 years), similar to what has been reported in other studies [18]. Recommendations to prevent diagnostic delays is to implement national Newborn Screening programmes for SMA, a move supported by 52% of our participants. Early diagnosis leading to early therapy initiation has been shown to result in substantial improvements in neurodevelopmental outcomes in Germany [19].

Majority of the participants from this study listed the lack of independent mobility and reduced physical activities followed by difficulty in self-care and personal grooming as the main impact of SMA on their physical limitations. These findings are similar to the results from a study in Spain where their participants listed difficulties in performing physical activities and self-care as the main impact SMA has on their lives [17]. It is not surprising that the participants in both studies are of Type 2 SMA, and they faced similar daily challenges despite living in different regions of the world.

Caregivers from this study also reported the high financial costs of caring for a PWSMA with many caregivers having to take up financial loans. This is further worsened by the need for one parent to resign from work to be a full-time caregiver. Even though healthcare services in Malaysian Government hospitals are affordable, the long waiting time and lack of specialized clinics to treat PWSMA has pushed caregivers to resort to seeking treatment in private healthcare centers. The costs for medical equipment, medications, physiotherapy, doctors’ visits add up to a very significant amount for SMA families. Various studies conducted in different regions of the world report similar financial pressure felt by the caregivers [7, 10, 12, 20]. This issue has also been raised by other rare disease groups in Malaysia, indicating a critical look at healthcare financing is needed for these vulnerable groups [21].

Another significant gap in care is that none of the participants had ever been referred to any palliative care specialist. A recent study in France illustrated the benefits of referring caregivers of PWSMA Type 1 for such consultations, as the level of care for respiratory, nutritional, pain and comfort management were enhanced, as well as their preparation to deal with end of life care and decisions [22].

Several PWSMA and their caregivers also shared about their experiences with access to education. While the Malaysian Education Ministry has drawn up a ‘No Child Left Behind’ initiative, mainly aimed at providing access to education for children living in poverty and with special needs, the acceptance of an PWSMA into a particular school is often left to the discretion of the school management, and there is no official training provided for teachers in mainstream schools in dealing with PWSMA. As such, school-life experiences were most commonly negative with only a small number experiencing a supportive school environment. School management often redirected parents to dedicated special needs schools, under the assumption that a child with physical disability would be better served there, often citing issues with infrastructure; despite parents feeling strongly that their children should enter mainstream curriculum as they did not have any learning disability. Although In 2017, the ministry announced measures to ensure all schools are disable-friendly by 2020, however this was only for improving special needs schools and not for mainstream schools to children with mainly physical needs such as PWSMA [23]. PWSMA also recounted bullying episodes or lack of support from teachers, similar to what has been reported by individuals with various other disabilities in Malaysia [24] suggesting systemic issues are present which need to be seriously addressed. Fostering an environment of inclusivity and non-discrimination can help to mitigate the negative attitude of Malaysians in general towards persons who are differently abled in order to achieve ‘education for all’ and inclusivity for disabled persons. A study by Galli et al. has demonstrated that people who maintain prolonged contact with persons with disabilities score positive attitudes towards them which emphasizes the fact that promoting awareness would sensitize both public and healthcare fraternity on communication, training and understanding of SMA [21].

PWSMA often face discrimination during work recruitment process and are not provided with equal job opportunities. Adult PWSMAs bemoaned the restrictive workplace environments and disabling barriers which are not conducive to navigate around i.e., wheelchair inaccessible and not equipped with disabled friendly toilets. This is despite a policy introduced by the Malaysian Government in 2010 whereby a quota of 1% employment of persons with disabilities in the public sectors was implemented [25]. This policy however is not adopted readily by the workforce as employers are not empathetic to providing flexible working hours and accessible infrastructure to enable them to contribute effectively. Stereotypes and prejudice towards people with disabilities is rampant within the work environment [26]. Employment benefits not only allows one to participate in daily activities, but also empowers one to be financially and socially independent. This lack of equal opportunity negatively affects the lives and livelihoods of PWSMA.

A clear theme from the interviews was the call for government to hasten the process of approving SMA drug therapy in Malaysia. The PWSMA and caregivers valued access to these therapies to improve motor function and breathing, as similarly reported in a study conducted in United States [27]. Access to disease modifying treatments for Malaysians comes only under the compassionate access pathways, or through clinical trials. Whilst the government has set aside an allocation for funding rare disease medication, the SMA therapies are not included. Proposals by a local think-tank group has offered possible solutions to financing treatments for rare diseases [28], which include trust funds by the private sector, allocation of public funds and regional collaboration to lower the cost of the treatments.

This study has several limitations; firstly, the small sample size did not allow for further analysis of associations with participants’ demographic data and their experiences. Secondly, the participants were recruited through a non-governmental organization (NGO), rather than through hospitals, which made it difficult to obtain full medical histories for further review. However, the NGO SMA contact list represented the best available database of SMA families in Malaysia during the study period. Furthermore, we would like to highlight that the involvement of the NGO also indicates the strength of community-driven research initiatives in pushing for more studies in these critical areas in the country and should be encouraged when clinical registries are not available. Thirdly, genetic diagnosis could not be confirmed for all our participants, as the availability of the SMA genetic test was not available for a few participants, either due to the lack of awareness of the test by the attending physician at the time or having been diagnosed many years prior when the test was not readily available. Out of the 26 cases in this study, 20 cases (76.9%) were genetically diagnosed. As for the remaining six, five were diagnosed by muscle biopsy (19.2%), one by electromyography (3.8%) along with their clinical symptoms’ progression. Thus, we acknowledge that non-SMA individuals might have participated in this study, although all who participated identified as having SMA or as a caregiver to one. Fourthly, recall bias may be present as participants recollections might have waned over time. However, the qualitative data provided rich information and reached data saturation to enable subsequent analysis.

In conclusion, this study has initiated an important step in raising the voices of PWSMA and their families, by highlighting a number of issues and their unmet needs. Moving forward, there is a need for a multidisciplinary team to provide a holistic care from diagnosis to treatment, rehabilitation, and psychosocial support. Transdisciplinary collaboration and open communication between all the stakeholders of various specialties focusing on patient and family centered goals are paramount [29]. Collaboration between established NGOs, healthcare providers and community support would be vital to aid with knowledge and capacity building focusing on how to care for PWSMA. There are various steps forward to changing perception towards PWSMAs, such as raising public awareness on SMA, improve accessibility to education without discrimination and ongoing health support from multidisciplinary teamwork. Sustained efforts from relevant stakeholders are required to bring about systemic change to empower those living with SMA in Malaysia.

## Data Availability

The datasets generated and analysed during this study are available from the corresponding author on reasonable requests.

## Appendix: Interview guides for qualitative research

A. **Experience related to diagnosis**
  1. Can you share your experience on how your/your love one’s diagnosis was made? What was the first symptom?
  2. How do you feel after you/your love one’s are diagnosed?
  3. Did the doctor explain the condition of SMA to you?
B. **Impact on life**
  4. How does SMA affect your/your love one’s life?
    - Intrapersonal (How you perceive yourself, your feelings)
    - Interpersonal (Relationship with your family, relatives, friends, colleagues etc.)
C. **Worries and Concern**
  5. What are some of the things you are most worried about?
    - Confronting premature death
    - Difficult treatment choices
    - Heartbreak and fear with loss of functional abilities
    - Coming to terms with lost expectations
    - Loss of sleep and stress
    - Social discomfiture and stigma
    - Limitations on social activities
    - Struggle to achieve Independence
    - Uncertainty and helplessness
    - Pressure on family finances
  6. How big of an impact has this item had on [your life/the life of the person you care for]? (Prompt: for each item that has been mentioned by participants/caregiver, ask them to rate each on a scale of 1–10 to their life.)
    - Eg: Client 1: client mentioned that social stigma and finance is the main issues, then:
    - Facilitator asks the client: In a scale of 1–10, please rate the impact of how financial issue has on to your life.
    - Or prepare a piece of paper (Likert scale template) to be used by researchers to ask participants (to rank from 1–10)
D. **Hopes and concerns for Treatment**
  7. [If not mentioned] do [you/the person you care for] do things/take steps to avoid being hospitalized? Tell me about that?
  8. Do [you/the person you care for] do things/take steps to avoid [respiratory events/things that might impact breathing]? Tell me about that?
  9. In the past 12 months, what kind of decline in motor function did [you/the person you care for] experience?
  10. Would a treatment that stops decline in motor function be meaningful to [you/the person you care for]?
  11. Would a treatment that improves your motor function be meaningful to [you/the person you care for]?
  12. What kind of improvement do [you/the person you care for] want to see from treatment?
